# Notes and News

**Published:** 1892-01-23

**Authors:** 


### NOTES AND NEWS.

COLLECTED AND COMMUNICATED.

WE have to state with great regret, in reference to the planning of the Hastings Hospital that Mr. Hy. Hall's name was inadvertently omitted in conjunction with Mr. Keith Young's.

A RECENT number of a military paper draws attention to the incomplete training of sanitary and medical departments of the English army. It remarks that: "The medical department is as much a part of a general's command as his commissariat or his engineers, and it is desirable that the officers who may on service have to lead our brigades and divisions should know what the working of almost the most necessary portion of their command is like." In India, our contemporary remarks, matters medical are kept much more up to the mark, and the medical department plays an important part on the occasions of sham fights, &c. In the German army the principal medical officer is taken into the general's confidence during manœuvres, and the scheme of the day's operations is confided to him; but even in the German army a complete system of communication between the medical department and the commanding officers is not maintained, and as the ambulance service is only partially rehearsed during field days, a perfect reliance cannot be placed on its operations during the greater confusion of real warfare.

THE conveyance and removal of the sick is a mere matter of ordinary drill, with a small amount of surgical training combined, and no deficiency need be in this respect. Where actual attendance on the sick in the hospital is concerned, a greater difficulty comes in. Orderlies must naturally be employed to supplement, where they do not actually occupy the place of the army Sisters. Here a greater amount of training is required and, unfortunately, not often to be had. Any army Sister will tell one that nursing in military hospitals is no light task. The idea that the Sister is a mere administrator of medicine, which is largely held, is a very mistaken one. The orderly does the rougher part of the work, it is true, including duties which fall to the wardmaid, for example, in civil hospitals; but when actual nursing is required, this falls solely and entirely to the lot of the Sister, who does not find that help at hand from the orderly that she could rely upon from the hands of assistant nurses in the wards of civil hospitals. A remedy has yet to be found. Things are better than they were, but under the light of modern civilisation and requirements they must be better still, and the medical department of the army must be no less responsive to the call of dicipline than any other part of it.

THE North-West London Hospital dances, which proved such a success last year, are announced to take place on February 15th and 25th, and March 9th. As before, they are being organised by Mrs. George Hening, of Hamilton Place, and Dr. Sibley, of Harley Street, the latter acting as honorary secretary. Among the list of patronesses we notice the names of the services of several well-known members of the medical profession, such as Mrs. Ernest Hart, Mrs. Symes Thompson, Mrs. Durham, Mrs. Blandford, Mrs. Grinley Hewitt, Mrs. William Flower, Mrs. Donald Hood, Mrs. Edmond Nash, Mrs. Stowers, and Mrs. Seymour Tuke. The dances are to take place in the whole suite of the Portman Rooms, and the Anglo-Hungarian band has been engaged for the series. The circulars also announce that the President (Mrs. Katherine St. Hill) and fellows of the Chirological Society will be present to give palmistry demonstrations for the benefit of the hospital funds.

THERE is an interesting little institution at North Malvern called St. John's (Free) Convalescent Home. It is for women and girls over 14, and was founded in January, 1889, by Mrs. J. W. Dyson Perrins, Davenham Bank, Malvern, for poor women and girls convalescent from serious illness or operation, for the purpose of affording change of air, good food, and rest. The time allowed in the home is from a fortnight to a month. Cases which are not admissible are those of recovery from contagious or infectious diseases, hopeless cases of consumption, cancer or scrofula, persons subject to epileptic or other fits, and to mental disorders, or immoral character, and those in such conditions of weakness as would disable them from dressing themselves or making their own beds. In each case a medical certificate is required, and a recommendation from a clergyman or some other competent person. The home is open to patients from all parts of England (excepting only G. F. S. members), and is unsectarian. There are twelve beds, four of which are placed at the disposal of the Worcester Infirmary. Every care has been taken to make the home in the highest and truest sense a home and not an institution; and the Lady Superintendent, Miss Agnes Batchelor, carries on the home in a most satisfactory manner. In connection with this work two of the nursing staff of St. John's House, Worcester, nurse the sick poor in their own homes, in Malvern Link, North Malvern, and Cornleigh parishes.

THE Edinburgh Royal Infirmary extension scheme was thoroughly discussed at the annual meeting on the 4th instant. The reasons for the enlargement are very weighty. Firstly, the number of patients during the last ten years has increased eighty per cent., and a record of applicants refused admission shows that the accommodation provided falls far short of the demand. Secondly, the present number of beds is too small for the adequate clinical instruction of the large number of students; and thirdly, a further demand upon the number is made by an application from the Association for the Medical Education of Women, requesting the admission of lady students to the infirmary. It appears to meet all these requirements, an additional 200 beds is necessary. It is proposed to distribute the beds in two pavilions. The ground floor of one of these, containing a medical and surgical ward, would be given up to the lady students and their teachers entirely. Additional nurses' accommodation will be necessary, and also some offices. Part of the site requisite is already purchased, and it is calculated that an outlay of £73,000 will be required. This sum is not collected or guaranteed, but the managers so stongly feel the necessity of the case, that subject to the approval of the contributors they are ready to carry on the scheme at once, trusting to public generosity and support. Hitherto, the subscriptions towards the Infirmary expenditure have been very liberal, and it is hoped that they will be still more so in future as an increased income of £10,000 will be required if the extension is carried out. Should it be so, the Edinburgh Royal Infirmary will be one of the most complete hospitals in the Kingdom.

